# Adaptive step size algorithm to increase efficiency of proton macro Monte Carlo dose calculation

**DOI:** 10.1186/s13014-019-1362-5

**Published:** 2019-09-09

**Authors:** Reto Kueng, Daniel Frei, Werner Volken, Fabian Stuermlin, Marco F. M. Stampanoni, Daniel M. Aebersold, Peter Manser, Michael K. Fix

**Affiliations:** 1 Division of Medical Radiation Physics and Department of Radiation Oncology, Inselspital, Bern University Hospital, and University of Bern, Bern, Switzerland; 2 Department of Physics, Swiss Federal Institute of Technology (ETH), Zurich, Switzerland; 3 Institute for Biomedical Engineering, University of Zurich and Swiss Federal Institute of Technology (ETH), Zurich, Switzerland

**Keywords:** Macro Monte Carlo, Proton therapy, Dose calculation

## Abstract

**Purpose:**

To provide fast and accurate dose calculation in voxelized geometries for proton radiation therapy by implementing an adaptive step size algorithm in the proton macro Monte Carlo (pMMC) method.

**Methods:**

The in-house developed local-to-global MMC method for proton dose calculation is extended with an adaptive step size algorithm for efficient proton transport through a voxelized geometry by sampling transport parameters from a pre-simulated database. Adaptive choice of an adequate slab size in dependence of material interfaces in the proton’s longitudinal and lateral vicinity is investigated. The dose calculation algorithm is validated against the non-adaptive pMMC and full MC simulation for pencil and broad beams with various energies impinging on academic phantoms as well as a head and neck patient CT.

**Results:**

For material interfaces perpendicular to a proton’s direction, choice of nearest neighbor slab thickness shows best trade-off between dosimetric accuracy and calculation efficiency. Adaptive reduction of chosen slab size is shown to be required for material interfaces closer than 0.5 mm in lateral direction. For the academic phantoms, dose differences of within 1% or 1 mm compared to full Geant4 MC simulation are found, while achieving an efficiency gain of up to a factor of 5.6 compared to the non-adaptive algorithm and 284 compared to Geant4. For the head and neck patient CT, dose differences are within 1% or 1 mm with an efficiency gain factor of up to 3.4 compared to the non-adaptive algorithm and 145 compared to Geant4.

**Conclusion:**

An adaptive step size algorithm for proton macro Monte Carlo was implemented and evaluated. The dose calculation provides the accuracy of full MC simulations, while achieving an efficiency gain factor of three compared to the non-adaptive algorithm and two orders of magnitude compared to full MC for a complex patient CT.

## Background

Over the past decade, dozens of new proton therapy facilities have entered clinical operation and hundreds of patients are treated with protons every day [1]. The main physical advantage of this modality compared to conventional photon radiotherapy is the steep distal dose fall-off of proton beams after a distinct dose peak (Bragg peak). This allows for more conformal dose distributions to the tumor, improved sparing of surrounding tissue and a lower integral dose. Due to the high conformity, uncertainties in the prediction of the dose distribution can however strongly affect the clinical outcome of the treatment [2]. It is therefore of high importance to accurately and precisely determine and deliver the dose to the patient. To achieve this, appropriate characterization of the physical interactions of protons in both the treatment head and the patient is necessary [3]. Pencil beam algorithms are still the most popular method for proton dose calculation, providing an estimate of the dose distribution in short computation time, however coming at cost of deteriorated accuracy due to simplifications in the applied physics models [4, 5]. Monte Carlo (MC) methods on the other hand are considered the gold standard for beam modeling and dose calculation in radiation therapy [6], accounting for physical interactions based on first principles and simulating the transport of primary as well as secondary particles. For proton therapy, packages like TOPAS [7] or GATE [8], which are both based on the Geant4 MC simulation toolkit [9], provide a framework for accurate dose calculation for both research and clinical applications. The drawback of such MC methods is usually the long computation times, which typically renders them unfeasible for implementation in daily clinical routine. Many techniques have been proposed to enhance efficiency of MC dose calculation by different variance reduction methods [10]. One approach is to use a local-to-global approach, which is a well-established method for electron dose calculation [11–13]. This macro MC (MMC) approach uses full MC simulations to pre-simulate local geometries and store probability distributions of transport parameters in a database, which is then accessed in the global simulation to propagate a particle through a patient geometry. The MMC concept was also applied to proton dose calculation, showing an efficiency gain of up to a factor of 200 compared to a full MC for homogeneous phantoms [14]. Other studies have investigated methods for fast proton dose calculation relying on pre-simulations under the term of track-repeating algorithms [15, 16]. These algorithms are based on the database of particle trajectories in water, which are used to determine the dose deposition in an inhomogeneous voxelized geometry by scaling the path length of each step and the angle between steps, according to the material and its mass density. While showing good agreement in the calculated dose with full MC simulations, processes such as hadronic interactions are not explicitly simulated using these methods. The proton MMC (pMMC) method on the other hand still performs particle transport on a history-by-history base through a medium, sampling energy loss, spatial deflection and hadronic interactions on a macro step basis. However, this pMMC method still has shortcomings when it comes to voxelized geometries such as a clinical patient computed tomography (CT) dataset, where the applicable macro step size is generally limited to the voxel size due to voxel-by-voxel changes of Hounsfield unit (HU) value.

The purpose of this work is to increase the efficiency of pMMC dose calculation in voxelized geometries by implementing a novel adaptive step size algorithm. Geant4 offers the option for dynamic step sizes, but step size variation is still on a microscopic level, as boundary crossings such as the change of material composition on a voxel border present a hard interface for the transport code. Another optimization in Geant4 method allows the skipping of boundaries in voxelized geometries, if the two regions on either side of the boundary consist of the same material, which in this particular application means the same HU value. In the case of inhomogeneous geometries like a patient CT, this would require a binning of HU values in order to benefit from this optimization. The presented adaptive step size algorithm for pMMC on the other hand aims for adaptive choice of macro steps covering multiple voxels of varying HU value without the need to cluster voxels in a pre-processing of the CT image. This allows proton transport in appropriately large macro steps while maintaining dosimetric accuracy without restrictions due to voxel size or binning of materials in the CT.

## Methods

### General concept of pMMC and database generation

**Fig. 1:**
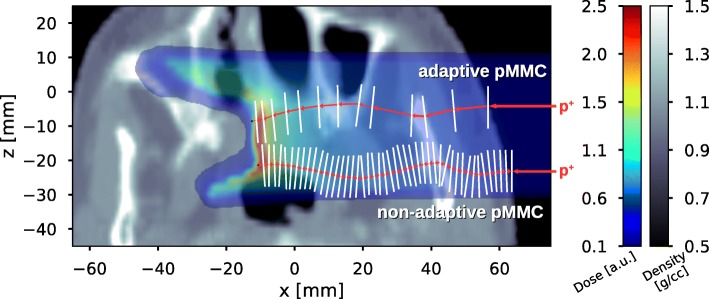
Schematic illustration of the proton MMC transport. A primary proton of certain energy is entering the voxelized geometry from the right. *Top:* With the adaptive pMMC, a slab of adequate size depending on energy and local environment of the proton is chosen and macro step parameters are sampled from the pre-simulated database. Smaller slabs are chosen for low proton energy and close proximity to material interfaces, larger slabs are chosen for a proton traversing a homogeneous environment. *Bottom:* With the non-adaptive pMMC, the macro step size in the patient is restricted by the voxel size in proton direction

The basic concept of the pMMC proton transport was introduced by Fix et al. [14]: To simulate the transport of a proton through a voxelized geometry, consecutive macro steps are applied by sampling transport parameters from probability distribution functions, which are pre-simulated and stored as histograms in a database. The parameters contain information about displacement, change in direction and energy loss throughout the macro step as well as information about occurrence and location of hadronic interactions. Figure 1 schematically illustrates the trajectory of a primary proton (only undergoing multiple scattering and ionization processes) for the adaptive as well as for the non-adaptive pMMC.

For database generation, slabs of different materials and thicknesses are simulated for a range of primary proton energies, such that a wide range of clinically relevant situations are covered. Geant4 version 10.4.p02 is used for the pre-simulation with the standard package (option 4) for the electromagnetic interactions, the hadron elastic physics list for the elastic interactions and the binary cascade physics list for the inelastic interactions. An overview of the pre-simulated materials and the corresponding CT ramp is shown in Table 1.

**Table 1:** Database materials defining the nodes of the CT ramp with corresponding mass stopping power ratio (mSPR) and empirical parameter for the continuous slowing down approximation *a*_CSDA_

Material	HU value	Density [g/cm ^3^]	mSPR	*a*_CSDA_ [cm/MeV ^2^]
Air	-1000	0.00125	0.88	0.765
Lung	-610	0.384	0.99	0.002640
Adipose tissue	-77	0.95	1.03	0.001019
Muscle	40	1.05	1.00	0.000963
Spongiosa	102	1.1	0.99	0.000920
Cortical bone	1524	1.92	0.89	0.000608
Teeth	3055	2.75	0.86	0.000445

The computation time of the pMMC dose calculation is dominated by the number of sampling procedures per proton trajectory. Therefore, in order to increase dose calculation efficiency, it is desired to minimize the number of macro steps by maximizing the slab size for each macro step. The slab size is however constrained by the accuracy loss occurring when using too large macro steps: Firstly, the kinetic energy of a proton determines its expected residual range, which limits the allowed macro step size. Secondly, material interfaces in the proton’s proximity break the condition of near-homogeneity requested by the local simulation and thus constrains the macro step size. Small slabs (i.e. a fine resolution of the proton path) are therefore favorable, if the proton has a low kinetic energy or is located in close proximity to a material interface. On the other hand, the largest available slabs are chosen, when the proton is traversing a homogeneous environment.

**Fig. 2:**
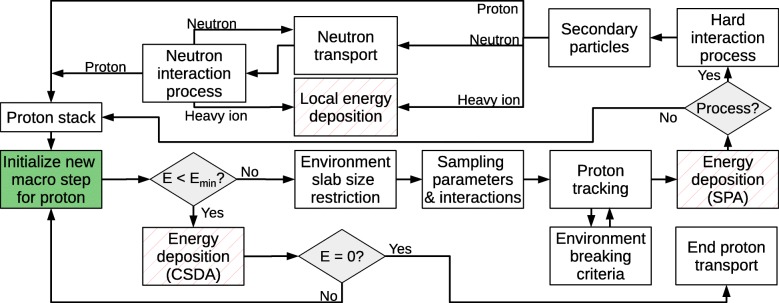
Flowchart of the conceptual logic of the proton transport in the pMMC method. Adaptive choice of slab size is performed (environment slab size restriction) and the macro step parameters are sampled from the database. The proton is transported (proton tracking) and deposits the sampled energy loss. If a hard interaction (elastic or inelastic hadronic interaction) was sampled (*Process?*), secondary protons and neutrons are transported and heavy ions deposit their energy locally

A flowchart of the proton transport in the pMMC algorithm is conceptually shown in Fig. 2. A new macro step is initialized for a proton at location ***x***_init_ and direction of motion ***u***_init_ in 3D space. The available slab sizes for the material of the proton’s current environment are extracted and the largest available slab for the current proton energy *E* is determined. If the kinetic energy of the proton is smaller than the lowest energy available in the database (E<*E*_min_), the proton deposits its energy via continuous slowing down approximation (CSDA) along its direction of motion ***u***_init_. If the proton energy is sufficiently large, environment slab size restriction is performed by an adaptive step size algorithm as described in the following subsection.

Macro step parameters and interaction processes are sampled from the database, yielding energy loss *ΔE*, exit position ***x***_out_ and exit direction ***u***_out_. If an elastic or inelastic hadronic interaction (hard interaction process) was sampled, the proton track ends at the point of interaction and the parameters (*ΔE*,***x***_out_,***u***_out_) are rescaled to the sampled interaction process point ***x***_proc_. The primary proton is then transported to the sampled end position ***x***_out_ or interaction process point ***x***_proc_ and energy is deposited via stopping power approximation (SPA, see following subsection). Finally, if an interaction process with secondary particles was sampled, secondary protons and neutrons are transported and heavy ions deposit their energies locally.

### Adaptive step size algorithm, proton tracking and energy deposition

Table 2 shows an overview of the improvements from the previous non-adaptive pMMC to the presented adaptive pMMC in this work. The aim of the transport algorithm is to allow a macro step to cross multiple voxels of varying HU values as long as the associated mixed material (as introduced by Fix et al. [14]) is maintained. Therefore, a material interface is defined as the boundary between two voxels where the respective HU values are separated by a node of the CT ramp (Table 1). Raytracing from ***x***_init_ along the initial proton direction ***u***_init_ is performed to detect the distance *d* to the next material interface. The slab size *s* closest to the distance *d* is chosen from the database to sample the parameters for the proton macro step.

**Table 2:** Features and capabilities of the non-adaptive (na-) and the adaptive (a-) pMMC

	na-pMMC	a-pMMC
Step size	Restricted by varying HU values	Adaptive choice of macro step
Energy deposition	Linear rescaling of deposited energy depending on covered fraction of macro step	Stopping power approximation over multiple voxels of varying HU value
Trajectory approximation	Hinge step, but macro step is interrupted at voxel boundary of varying HU value	Hinge step at 30% of macro step size, passes multiple voxels of varying HU value
Dose calculation accuracy	within 1% or 1 mm compared to full MC	
Efficiency: homogeneous geometries	very good	
Efficiency: inhomogeneous geometries	limited	very good

As raytracing along ***u***_init_ is not sensitive to potential lateral material inhomogeneities, macro step size restriction by material interfaces lateral to the initial proton direction ***u***_init_ is individually analyzed. To investigate the impact of lateral inhomogeneities, infinitesimal pencil beams impinging at lateral distances of 0.1, 0.2,..., 1.0 mm to a material interface are simulated without step size restriction. Dosimetric differences to Geant4 are quantified to determine the critical lateral distance under which the macro step size requires restriction to maintain dosimetric accuracy.

The softened definition of material interfaces as introduced above allows for a macro step to cross multiple voxels of varying HU values, which might potentially compromise dosimetric accuracy. Macro step size restriction due to HU value variation within a mixed material is investigated by simulating proton beams in phantoms of sole mixed materials, but layer-by-layer (1 mm) varying HU value. Dose calculations with the adaptive pMMC without macro step size restriction are compared to Geant4 calculations and dose differences are quantified in order to evaluate the impact of varying HU values within a macro step.

#### Proton tracking and hinge step

**Fig. 3:**
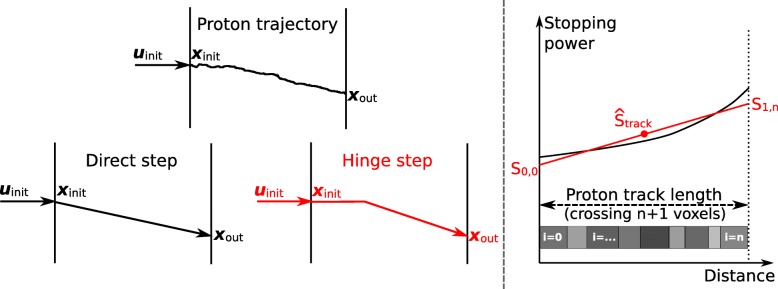
Proton track through a macro step. *Left:* Illustration of a possible realistic proton trajectory and the approximation as a direct and hinge step. *Right:* Qualitative trend of the proton stopping power and the used approximation for dose deposition in the pMMC method. Over the distance of the proton track, multiple voxels of varying HU values can be crossed in oblique angles, yielding different cutting lengths of proton track and voxels *i*=1,…,*n*

In order for the adaptive step size algorithm to be dosimetrically accurate, appropriate distribution of the sampled energy loss *ΔE* over the crossed voxels in a macro step is essential. Note that the pre-simulated slabs do not contain information about the trajectory within the slab. Crossing the voxels from the initial position ***x***_init_ to the sampled end position ***x***_out_ in a straight line (direct step) is an oversimplification yielding inaccurate dose distributions. Instead, the proton trajectory is approximated with a hinge step, which is first continuing in the initial proton direction of motion and after 30% of the macro step size turns towards the sampled end position. While in general the position of the hinge could be described as a function of proton energy and material, the value of 30% was found to be a sensible approximation for the range of investigated proton energies and materials. The approximative trajectory of the proton in a macro step is referred to as the *proton track*. Figure 3 (left) depicts the concept of the hinge step. While tracking the proton to the sampled end position, the environment breaking criterion is checked and the proton track ends if the proton track crosses a material interface.

#### Energy deposition via stopping power approximation (SPA)

The sampled energy loss is distributed to the crossed voxels *i* of the proton track according to

1 $$ E_{\text{Dep},i}(d_{i},\rho_{i}, \textrm{mSPR}_{i}) = d_{i} \cdot\frac{(S_{0,i} + S_{1,i})}{2}\cdot \frac{\rho_{i}\cdot \textrm{mSPR}_{i}}{\hat{\rho}\cdot\widehat{\textrm{mSPR}}},   $$

where *d*_*i*_ is the cutting length of the proton track and voxel *i* and *S*_[0,1],*i*_ are the stopping power at the entry and exit points of the proton track in voxel *i*. Furthermore, *ρ*_*i*_ is the physical density and mSPR_*i*_ is the mass stopping power ratio of the material in voxel *i*, which are normalized by the mean density $\hat {\rho }$ and the mean mass stopping power ratio $\widehat {\textrm {mSPR}}$ over the proton track. This allows accurate energy deposition on a voxel level, although the proton track can cover multiple voxels of varying HU values. The stopping power at the starting point of the proton track

2 $$ S_{0,0} = \hat{S}_{\text{track}} - \frac{d_{\text{track}}}{2}\cdot\left(\frac{\Delta S}{\Delta x}\right)_{\text{track}},  $$

where $\hat {S}_{\text {track}} = \Delta E / d_{\text {track}}$ is the mean stopping power along the proton track, given by the sampled energy loss *ΔE* divided by the length of the proton track *d*_track_. A linear increase of the stopping power over the proton track is semi-empirically approximated:

3 $$ \left(\frac{\Delta S}{\Delta x}\right)_{\text{track}} = {0.8}\ \mathrm{MeV\ cm^{-2}} \cdot \frac{\left(\frac{\hat{S}_{\text{track}}}{[\mathrm{MeV/cm}]}\right)^{2} - 4\left(\frac{\hat{\rho}\cdot\widehat{\textrm{mSPR}}}{[\text{g/cm}^{3}]}\right)^{2}}{\frac{E - \frac{\Delta E}{2}}{[\text{MeV}]}}  $$

The stopping power at the exit point of voxel *i* of the proton track is then determined by

4 $$ S_{1,i} = S_{0,i+1} = S_{0,i} + d_{i} \cdot \left(\frac{\Delta S}{\Delta x}\right)_{\text{track}}.  $$

Figure 3 (right) shows a schematic illustration of the stopping power approximation.

#### Energy deposition via continuous slowing down approximation (CSDA)

For low proton energies of ≲10 MeV, the remaining proton energy is deposited via CSDA along its initial direction of motion ***u***_init_. The residual range is approximated by

5 $$ {}\begin{aligned} R_{\textrm{CSDA}}\! = \int_{0}^{E} \frac{1}{S} \mathrm{d}E' = \int_{0}^{E} \frac{1}{\left(\frac{\mathrm{d}E'}{\mathrm{d}x}\right)} \mathrm{d}E' \approx a_{\textrm{CSDA}}E(E+{2}\text{MeV}),  \end{aligned}  $$

where the parameter *a*_CSDA_ [cm/MeV ^2^] is a material dependent value fitted to NIST PSTAR data [17] and stored in the slab database for all materials (see Table 1) and *E* [MeV] is the proton’s kinetic energy. For mixed materials, interpolation between the *a*_CSDA_ values of the database materials according to the local HU value is performed.

### Validation

In order to benchmark the adaptive step size algorithm for accuracy and efficiency, academic cases as well as a head and neck patient case are studied by comparison of integral depth dose curves and lateral contour plots against the results of the non-adaptive pMMC algorithm and full MC calculations with Geant4. Gamma evaluation [18] is additionally performed in order to assess an additional parameter to quantify dosimetric differences between the adaptive pMMC algorithm and Geant4. Mono-energetic (100, 150, 200 and 250 MeV) infinitesimal pencil beams and broad beams (4×4 cm ^2^) are applied to the validation cases.

As discussed by Schümann et al. [19], Geant4 offers several navigation algorithms for voxel geometry parametrization that differ in memory usage and performance. For this work, the G4PhantomParameterisation is used for the validation simulations with Geant4. Following the findings of this work, the boundary skipping option is explicitly turned off as no performance increase can be expected for the investigated cases and the skipping option showed the danger of wrong dose deposition [19].

**Fig. 4:**
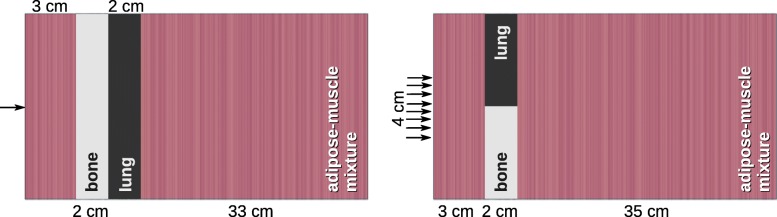
Academic cases used for validation: Mono-energetic proton beam impinging on a mixture of adipose and muscle tissue with layer-by-layer varying HU value and lung and bone inhomogeneities in series (left) and adjacent to each other (right), establishing a material interface perpendicular and parallel to the initial proton direction, respectively

**Academic cases** Academic cases are designed to challenge the accuracy of the adaptive step size algorithm by forcing proton beams to pass material interfaces between high and low density materials and to propagate through a mixed medium of varying HU value. The phantoms contain *x*×*y*×*z*=100×100×400 voxels with dimensions of 0.2×0.2×0.1 cm ^3^. They consist of a mixture of adipose and muscle tissue with layer-by-layer varying HU value between -77 and 40 (physical density between 0.95 and 1.05 g/cm^3^). Lung and bone inhomogeneities in series (Fig. 4 left) and adjacent to each other (Fig. 4 right) are introduced, establishing material interfaces perpendicular and parallel to initial proton direction, respectively. The beams are impinging on the phantoms (broad beam: centered) at (*x*,*y*,*z*)=(0,0,0) [cm] in z-direction.

**Head and neck case** For the head and neck patient case, a broad beam is applied centered at (*x*,*y*,*z*)=(10.0,−14.0,−1.0) [cm] in negative x-direction and is containing *x*×*y*×*z*=236×144×118 voxels with dimensions of 0.1×0.2×0.2 cm ^3^.

**Efficiency** The efficiency *ε* of the MC transport algorithms is defined as *ε*:=(*T*·*σ*^2^)^−1^, where *T* is the calculation time to achieve the statistical uncertainty *σ* in the dose distribution. For the purpose of establishing a comparison of the efficiency *ε* of the three different algorithms (adaptive and non-adaptive pMMC, Geant4), all simulations for the benchmarking are performed on a single core of the same machine and with an equal number of 10^6^ (pencil beams) and 10^7^ (broad beams) simulated primary protons. This yields a statistical uncertainty of the dose distribution within the phantom below 1% (1 standard deviation) for all MC calculations, as assessed by a history-by-history (pMMC) and batch (Geant4) uncertainty estimation. The utilized working station is a Dell Precision T5600 equipped with an Intel Xeon CPU E5-2620 (2.00 GHz, 15 MB cache). The efficiency gain is calculated by comparing the single core runtime of the pMMC algorithms versus the full MC with Geant4 for the introduced cases.

## Results

### Adaptive step size algorithm

**Fig. 5:**
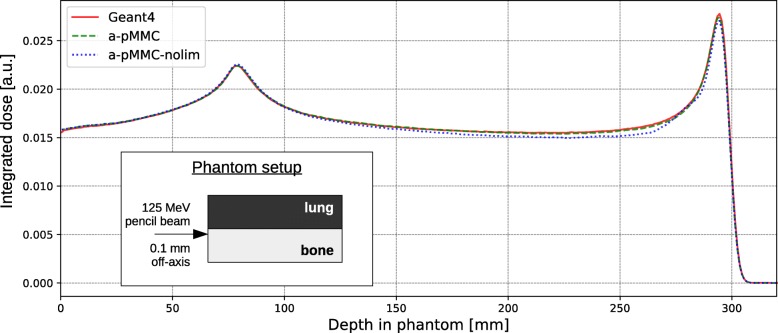
Investigation of lateral material interface. Integrated depth dose curves for a mono-energetic proton pencil beam (125 MeV) impinging parallel to a material interface at a lateral distance of 0.1 mm. Lines are for the adaptive pMMC (dashed), adaptive pMMC without step size restriction (dotted) and full Monte Carlo with Geant4 (solid)

Macro step size restriction by material interfaces lateral to the proton’s direction of motion ***u***_init_ is found to be necessary to maintain dosimetric accuracy. For the investigated scenario, lateral distances from the infinitesimal pencil beam to the interface smaller than 0.5 mm reveal a drop in dosimetric accuracy. Exemplarily, Fig. 5 shows integrated depth dose curves for a pencil beam at a lateral distance of 0.1 mm to a bone/lung material interface with and without step size restriction. While the adaptive pMMC with restriction shows agreement within 1% dose difference, dose differences exceed 5% if no step size restriction is applied. For this example, macro step size restriction comes at the cost of a factor of 2 in calculation time. Nonetheless, to ensure dosimetric accuracy, a threshold value of 0.5 mm lateral distance to a material interface is defined below which the smallest available slab size is selected for the macro step.

In contrast, variation of HU values within any mixed material is found not to influence the accuracy of the adaptive pMMC proton transport. For all investigated cases of layer-by-layer varying HU value, dose difference and distance to agreement are below 1% or 1 mm without macro step size restriction.

### Academic cases

**Fig. 6:**
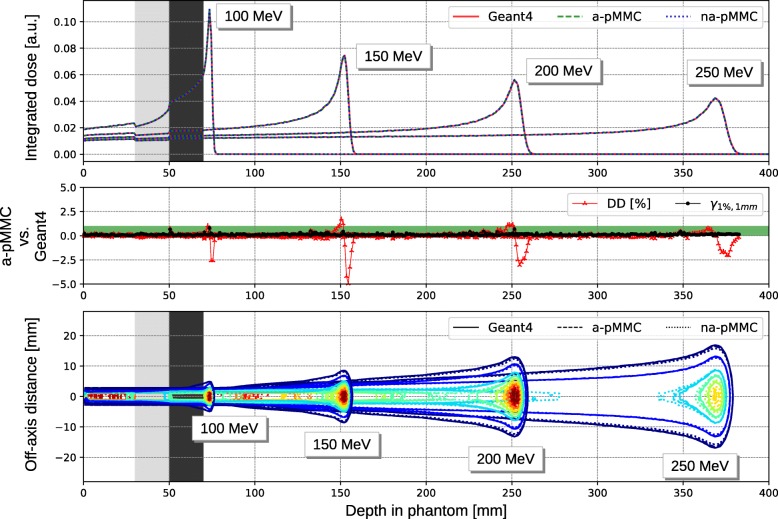
Results of the academic phantom with layered inhomogeneities. Integrated depth dose curves (top) with corresponding dose difference (DD) and 1D gamma evaluation (middle) and contour plot (bottom) for the academic phantom with lung and cortical bone in layers for mono-energetic proton pencil beams of 100 MeV, 150 MeV, 200 MeV and 250 MeV. Lines are for the adaptive pMMC (dashed), non-adaptive pMMC (dotted) and Geant4 (solid). The isodose lines indicate 10%, 20%, 40%, 50%, 60%, 70%, 80%, 90%, 95% and 99% of the respective reference dose maximum

Figure 6 shows the integrated depth dose curves (top) and the contour plot (bottom) for mono-energetic infinitesimal pencil beams impinging on the academic case with lung and cortical bone inhomogeneities in series. As expected, a decreased dose can be observed in cortical bone, as the higher effective Z value yields a lower mass stopping power due to increased ionization potential. For all investigated energies, the depth dose curves agree between the adaptive pMMC, the non-adaptive pMMC and Geant4 calculations. Dose differences are below 1% between adaptive pMMC and Geant4 for all depths except for regions very close to the Bragg peak with steep dose gradients, where dose differences up to 5% can be observed. At these points however, distance to agreement is well below 1 mm and therefore, 1D-Gamma passing rate is 100% for all energies. The contour plot shows equally high agreement between the dose distributions. Isodose lines indicating 10%, 20%, 40%, 50%, 60%, 70%, 80%, 90%, 95% and 99% of the respective reference dose maximum show no observable loss in dosimetric accuracy for all energies.

**Fig. 7:**
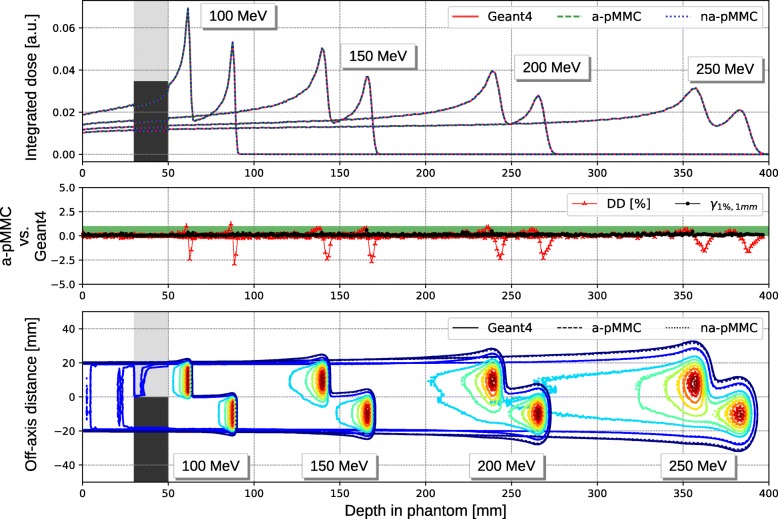
Results of the academic phantom with adjacent inhomogeneities. Integrated depth dose curves (top) with corresponding dose difference (DD) and 1D gamma evaluation (middle) and contour plot (bottom) for the academic phantom with lung and cortical bone adjacent to each other for mono-energetic proton 4×4 cm^2^ broad beams of 100 MeV, 150 MeV, 200 MeV and 250 MeV. Lines are for the adaptive pMMC (dashed), non-adaptive pMMC (dotted) and Geant4 (solid). The isodose lines indicate 10%, 20%, 40%, 50%, 60%, 70%, 80%, 90%, 95% and 99% of the respective reference dose maximum

Figure 7 shows the integrated depth dose curves (top) and the contour plot (bottom) for mono-energetic 4×4 cm^2^ broad beams impinging on the academic case with lung and cortical bone inhomogeneities adjacent to each other. Again, the integrated depth dose curves for all calculation methods are in agreement. For all energies, dose differences are smaller than 1%, with exception of several points in proximity to the Bragg peak, where dose differences up to 2.5% are observed. Distance to agreement at these evaluation points is below 1 mm, yielding a 1D-Gamma passing rate of 100% for all energies. The lateral dose distribution illustrated by the contour plot show no relevant dosimetric differences between the three calculation methods.

### Head and neck case

**Fig. 8:**
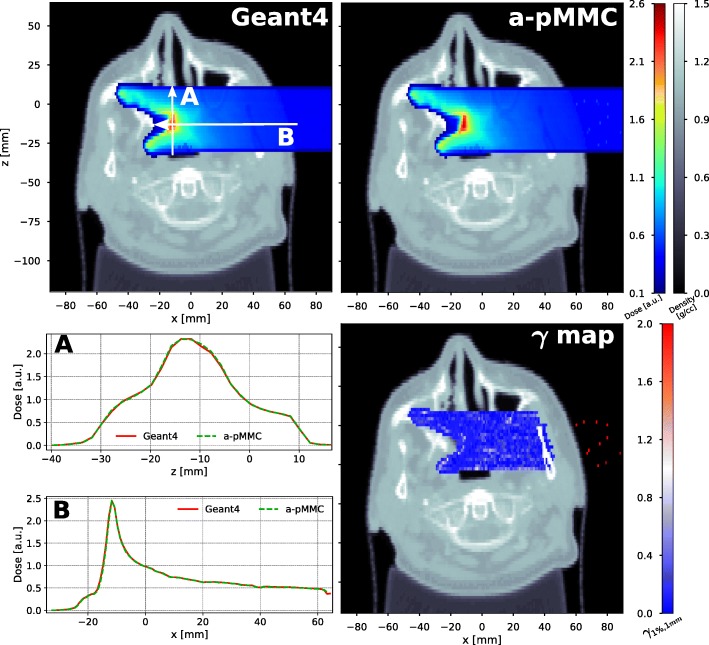
Results of the patient CT case. Transversal cut of the head and neck patient CT showing dose color washes for a mono-energetic proton 4×4 cm^2^ broad beam of 100 MeV calculated with Geant4 (top left) and the adaptive pMMC (top right). Dose profiles as indicated by the white arrows are shown (bottom left) and the result of a 3D-Gamma evaluation with a 1% (global) and 1 mm criterion (20% lower cutoff) is presented for the corresponding slice (bottom right)

Figure 8 shows dose washes and dose profiles of a 100 MeV broad beam impinging on the head and neck patient case as well as the *γ* map for a single transversal CT slice. Dose distributions are in excellent agreement between the adaptive pMMC calculation and the full MC Geant4 calculation. A three dimensional Gamma evaluation with a 1% global dose difference criterion and a 1 mm distance to agreement criterion with a lower dose cutoff at 20% of the reference dose maximum yields a 99.3% passing rate. The majority of the points failing the Gamma criterion are located in the air proximal to the patient outline, which can be observed in the *γ* map in Fig. 8 (right). Omitting air voxels in the evaluation, the Gamma passing rate increases to 99.97%. No gamma values are indicated in the bony region close to the surface due to the dose being just below the 20% threshold. This dose decrease in high-Z materials is explained above for the academic case.

### Efficiency

Table 3 shows the efficiency gain of the adaptive step size algorithm compared to the non-adaptive pMMC and full MC calculations with Geant4. For the academic cases, an efficiency gain in the range of 183 to 284 between the adaptive pMMC and Geant4 calculations is found. Compared to the non-adaptive pMMC, the efficiency gain is the order of a factor 5. For the patient CT, the efficiency gain is two orders of magnitude (factor 100) for the adaptive pMMC with respect to Geant4. Compared to the non-adaptive pMMC, the efficiency gain is up to a factor of 3.4.

**Table 3:** Efficiency gain factor of the adaptive pMMC versus the non-adaptive (na) pMMC and full Monte Carlo calculation with Geant4 (G4)

	100 MeV		150 MeV		200 MeV		250 MeV	
Phantom	na	G4	na	G4	na	G4	na	G4
Lung & cortical bone in series	3.4	182.9	4.8	233.7	5.0	250.2	5.6	284.0
Lung & cortical bone adjacent	4.5	217.7	5.3	248.7	5.2	256.7	5.3	254.1
Head and neck case	2.9	115.5	3.4	134.8	3.4	142.0	3.4	145.3

## Discussion

The implementation of an adaptive step size algorithm for the pMMC method shows substantial benefits in the efficiency of the dose calculation without observable loss in dosimetric accuracy. For the academic and patient validation cases, all results show very good agreement between the adaptive pMMC and full MC calculations with Geant4. Compared to the non-adaptive pMMC, dosimetric accuracy is in fact even improved. This can be attributed to the fact that the adaptive pMMC has a more advanced implementation of the energy deposition over a macro step. As a consequence, macro steps that are intercepted in the proton tracking due to a material interface are more accurately described by the adaptive pMMC compared to the non-adaptive transport algorithm. In terms of efficiency gain, a speed-up factor of 3-5 is found for the adaptive pMMC compared to the non-adaptive algorithm, which can be considered a substantial improvement of the algorithm. Compared to Geant4 calculations, efficiency gain is at least a factor of 100 and ranging up to a factor of 284 for the academic cases at high energy. The energy dependence of the gain factor can be explained by the fact that for higher initial energy of the proton beam, the adaptive algorithm can profit from a high number of large macro steps, whereas for lower energy protons step size is already restricted by kinetic energy in shallower depths. It should be noted that the adaptive step size algorithm for pMMC does not require any kind of pre-processing or binning of materials in the CT image. Instead, the algorithm is using raytracing to identify feasible macro steps on the fly during proton transport. Therefore, no coarsening of the dose grid resolution is required and proton transport efficiency is only slightly depending on grid resolution due to the raytracing needed in the environment slab size restriction and the proton tracking. However, the pMMC method is still susceptible to uncertainties due to CT calibration and conversion to tissue (described by Paganetti et al. [20]), as the definition of the CT ramp with corresponding materials (Table 1) strongly influences the generation of the database and thus the pMMC dose calculation. This could be refined by defining more database materials such as suggested by Schneider et al. [21], which would narrow HU value bands of mixed material and thus generally constrain the adaptive pMMC transport to smaller macro steps. In this work, we validate the adaptive pMMC algorithm against Geant4 simulations for simple pencil and broad beams. Earlier studies have shown good agreement of Geant4 based dose calculation with measurements [7, 22–25]. Still, for use in a clinical setting, the pMMC dose calculation algorithm needs to be commissioned to a beam model for passive scattering or spot scanning treatment technique. The presented efficiency gain in this work is reported for running the dose calculation on a single CPU core. Effective calculation time can be optimized by parallelization on multiple CPU cores. Further parallelization of the MC code to run on a graphics processing unit (GPU) as reported in other studies [26–28] promises a further boost in simulation performance. A designated implementation of pMMC optimized for running on a GPU is expected to reduce the effective calculation time of a proton therapy treatment plan to a acceptable level for use in inverse optimization.

## Conclusion

The in-house developed MMC method for proton dose calculation was extended with an adaptive step size algorithm for improved efficiency in voxelized geometries, such as patient CTs. The calculation efficiency was shown to be improved by a factor of up to 5.6 for the academic cases and up to 3.4 for the patient case with respect to the non-adaptive algorithm and two orders of magnitude with respect to full MC, while keeping dosimetric accuracy.

## Data Availability

The datasets used and analysed during the current study are available from the corresponding author on reasonable request.
